# Increased expression of six-large extracellular vesicle-derived miRNAs signature for nonvalvular atrial fibrillation

**DOI:** 10.1186/s12967-021-03213-6

**Published:** 2022-01-03

**Authors:** Panjaree Siwaponanan, Pontawee Kaewkumdee, Wilasinee Phromawan, Suthipol Udompunturak, Nusara Chomanee, Kamol Udol, Kovit Pattanapanyasat, Rungroj Krittayaphong

**Affiliations:** 1 Siriraj Center of Research Excellence for Microparticle and Exosome in Diseases, Department of Research and Development, Faculty of Medicine Siriraj Hospital, Mahidol University, Bangkok, Thailand; 2 Division of Cardiology, Department of Medicine, Faculty of Medicine Siriraj Hospital, Mahidol University, Bangkok, Thailand; 3 Division of Clinical Epidemiology, Department of Research and Development, Faculty of Medicine Siriraj Hospital, Mahidol University, Bangkok, Thailand; 4 Department of Pathology, Faculty of Medicine Siriraj Hospital, Mahidol University, Bangkok, Thailand; 5 Department of Preventive and Social Medicine, Faculty of Medicine Siriraj Hospital, Mahidol University, Bangkok, Thailand

**Keywords:** Large extracellular vesicles, miRNA, Biomarkers, Patients, Atrial fibrillation

## Abstract

**Backgrounds:**

Non-valvular atrial fibrillation (AF) is the most common type of cardiac arrhythmia. AF is caused by electrophysiological abnormalities and alteration of atrial tissues, which leads to the generation of abnormal electrical impulses. Extracellular vesicles (EVs) are membrane-bound vesicles released by all cell types. Large EVs (lEVs) are secreted by the outward budding of the plasma membrane during cell activation or cell stress. lEVs are thought to act as vehicles for miRNAs to modulate cardiovascular function, and to be involved in the pathophysiology of cardiovascular diseases (CVDs), including AF. This study identified lEV-miRNAs that were differentially expressed between AF patients and non-AF controls.

**Methods:**

lEVs were isolated by differential centrifugation and characterized by Nanoparticle Tracking Analysis (NTA), Transmission Electron Microscopy (TEM), flow cytometry and Western blot analysis. For the discovery phase, 12 AF patients and 12 non-AF controls were enrolled to determine lEV-miRNA profile using quantitative reverse transcription polymerase chain reaction array. The candidate miRNAs were confirmed their expression in a validation cohort using droplet digital PCR (30 AF, 30 controls). Bioinformatics analysis was used to predict their target genes and functional pathways.

**Results:**

TEM, NTA and flow cytometry demonstrated that lEVs presented as cup shape vesicles with a size ranging from 100 to 1000 nm. AF patients had significantly higher levels of lEVs at the size of 101–200 nm than non-AF controls. Western blot analysis was used to confirm EV markers and showed the high level of cardiomyocyte expression (Caveolin-3) in lEVs from AF patients. Nineteen miRNAs were significantly higher (> twofold, *p* < 0.05) in AF patients compared to non-AF controls. Six highly expressed miRNAs (miR-106b-3p, miR-590-5p, miR-339-3p, miR-378-3p, miR-328-3p, and miR-532-3p) were selected to confirm their expression. Logistic regression analysis showed that increases in the levels of these 6 highly expressed miRNAs associated with AF. The possible functional roles of these lEV-miRNAs may involve in arrhythmogenesis, cell apoptosis, cell proliferation, oxygen hemostasis, and structural remodeling in AF.

**Conclusion:**

Increased expression of six lEV-miRNAs reflects the pathophysiology of AF that may provide fundamental knowledge to develop the novel biomarkers for diagnosis or monitoring the patients with the high risk of AF.

**Supplementary Information:**

The online version contains supplementary material available at 10.1186/s12967-021-03213-6.

## Introduction

Nonvalvular atrial fibrillation (AF) is the most common sustained cardiac arrhythmia. AF results from electrophysiological abnormalities and alteration of atrial tissues, which promote the formation and propagation of abnormal electrical impulses. The prevalence of AF is gradually increasing worldwide, especially among older adults, and this is causing important health, social, and economic problems, and increases the risk of stroke, heart failure, morbidity, and disability [1].

Diagnosis and prognosis in AF are based on evaluation of symptoms, monitoring of irregular rhythm, and assessment of cardiac anatomical structure and function; however, most AF patients are asymptomatic and effective long-term monitoring is challenging. Therefore, the identification of blood-derived biomarkers may help physicians evaluate AF risk and severity.

The development and propagation of AF are influenced by electrical and structural remodeling of the atria. Predisposing factors associated with electrophysiological abnormalities in AF are aging, cardiovascular risk factors (e.g., hypertension, diabetes, dyslipidemia, heart failure, obstructive sleep apnea, and obesity), genetic mutation, and dysregulation of ion channels and transporter expression [2]. Current studies have focused a great deal of attention on gene regulatory mechanisms, particularly microRNA [3]. MicroRNAs (miRNAs) are small non-coding ribonucleic acid (RNA) molecules that average 22 nucleotides in length that mediate post-transcriptional gene regulation by sequence-specific inhibition of target mRNA translation. They are essential in a variety of biological processes, including proliferation, differentiation, apoptosis, and metabolism [4]. More than 2500 miRNAs have been identified in the human genome, and more than 1500 miRNAs have had their gene regulatory functions formally defined [5]. MicroRNAs that are released into extracellular fluids (e.g., plasma, urine, saliva), are called circulating miRNAs or extracellular miRNAs. Three pathways of miRNA secretion have been reported, including incorporation with high-density lipoprotein (HDL), binding with Argonaut 2 (Ago2), and encapsulation in extracellular vesicles [6].

Extracellular vesicles (EVs) are lipid bound vesicles that are released by many cell types into the extracellular space. EVs are found in various body fluids such as blood, urine, cerebrospinal fluid, and saliva. Increasing evidence suggests that EVs act as a vehicle to transfer genetic materials in cell-to-cell communication [7]. MicroRNAs derived from extracellular vesicles (EV-miRNAs) play important roles in both normal homeostasis and pathophysiology in many diseases, including cardiovascular diseases (CVDs) [8, 9]. EV-miRNAs have been described as a selective packaging mechanism that parental cells sort out a set of miRNAs into EVs for secretion to target cells [10, 11]. International Society for Extracellular Vesicles (ISEV) has suggested to classify EVs based on difference in size as small EVs (< 100 nm or < 200 nm) and medium/large EVs (> 200 nm) [12]. Small EVs (sEVs), also referred to as exosomes, are smallest types of EVs (30–150 nm) and released by inward budding of multivesicular bodies (MVBs) from plasma membrane into the extracellular space. Large EVs (lEVs), also called microvesicles (MVs) or microparticles (MPs), are secreted by outward budding from plasma membrane during cell activation or cell stress and have a size ranging from 100 to 1000 nm.

Expression of EV-miRNAs has been shown to be associated with AF and reflect pathophysiology of AF [13–15]. However, all previous studies focused on sEV-miRNAs, there are no evidences of the relationship between lEV-miRNAs and AF. lEV-miRNAs have been reported to be diagnostic and prognostic biomarkers in several diseases. Jansen F, et al. reported that increased expression of miR-126 and miR-199a in MPs, but not freely circulating miRNA expression, predicts the occurrence of cardiovascular events in patients with stable coronary artery disease [16]. miR-129-5p isolated from plasma MVs was demonstrated to be a sensitive and specific biomarker for heart failure (HF) in univentricular heart disease [17]. In addition, MP miR-124a and miR-150, which were found to be more abundant in obese subjects compared to normal weight controls, were found to be significantly associated with inflammation and vascular function in obesity [18]. As a consequence, lEV-miRNAs are emerging as attractive biomarkers due to their stability and easy detection in biofluids. lEV-miRNA detection can be sensitive, predictive, specific, and noninvasive, which are all characteristics of an ideal biomarker [19]. The aim of this study was to investigate for differences in lEV-miRNA expression profiles between AF patients and non-AF controls. Subsequently, lEV- miRNAs of interest were validated using droplet digital polymerase chain reaction (ddPCR), and bioinformatics analysis was used to predict miRNA target genes and their functional pathways. The results of this study revealed that the expression signature of six lEV-miRNAs that reflect pathophysiology of AF.

## Material and methods

### Study population and subject enrollment

AF patients were recruited from the Division of Cardiology, Department of Medicine and the control subjects were recruited from Department of Preventive and Social Medicine, Faculty of Medicine Siriraj Hospital, Bangkok, Thailand during November 2019 to December 2020. The protocol for this study was approved by the Siriraj Institutional Review Board (SIRB) (COA no. Si 489/2019), and complied with the principles set forth in the Declaration of Helsinki (1964) and all of its subsequent amendments. All study participants provided written informed consent prior to inclusion. Forty-two AF patients (3 new-onset AF, 28 paroxysmal AF, 8 persistent AF, and 3 permanent AF) and 42 age- and sex-matched non-AF controls were enrolled. All participants underwent a thorough historical investigation and 12-lead electrocardiography (ECG) to confirm cardiac rhythm. Twelve AF patients and 12 non-AF controls were enrolled for the discovery phase, and the other 30 AF patients and 30 non-AF controls were included in the validation phase. Non-AF controls had no history of CVDs, no history of atrial arrhythmias, and no current cardiovascular treatment. No study participants had history of myeloproliferative disorders, thrombocytopenia, ischemic stroke within the previous 3 months, malignancy, rheumatic mitral valve disease, acute infectious or inflammatory disease, or pregnancy.

### Blood processing and lEV isolation

Peripheral blood that was collected into BD Vacutainer® K2EDTA Plus Blood collection tubes (BD Biosciences, Franklin Lakes, NJ, USA), was centrifuged at 1500 ×*g* for 15 min to isolate platelet poor plasma (PPP), and stored at − 80 °C until analysis. Blood specimens were processed within 4 h after blood draw. lEV isolation was performed as previously described [20]. Briefly, PPP was centrifuged at 17,000 ×*g* for 2 min at 4 °C to remove remaining platelets, and the lEVs were pelleted by centrifugation at 17,000 ×*g* for 45 min at 4 °C. lEV pellets were washed with filtered phosphate-buffered saline (PBS) prior to resuspension with 100 μl of fresh PBS.

### Nanoparticle tracking analysis (NTA)

lEV concentration and size distribution were measured using a NanoSight NS300 (Malvern Panalytical, Malvern, UK) equipped with a 488 nm laser. lEV suspension was diluted (1:100–1:200) in filtered PBS. Samples were analyzed under constant flow conditions (flow rate: 30) at 25 °C, and were captured with a camera level of 13–14 using NTA software version 3.4 (Malvern Panalytical). Five independent measurements (60 s each) were obtained for each sample. Data are reported as mean ± standard deviation (SD).

### Transmission electron microscopy (TEM)

lEV suspensions were fixed in 2% glutaraldehyde (Sigma-Aldrich Corporation, St. Louis, MO, USA) in PBS for 30 min at 4 °C, and then absorbed onto 200 mesh copper grids with carbon-coated formvar film for 15 min. The grids were negatively stained with 2% uranyl acetate (w/v) for 3 min, and then the excess liquid was removed by blotting with filter paper. Grids were imaged under a transmission electron microscope (JEM-1230; JEOL Ltd, Tokyo, Japan) at 100 kV.

### Flow cytometry

Five microliters of each lEV suspension was analyzed using a CytoFLEX S flow cytometer (Beckman Coulter Life Sciences, Indianapolis, IN, USA) and CytoExpert analysis software (Beckman Coulter Life Sciences). lEV gate was indicated by size calibration beads (Spherotech, Inc., Lake Forest, IL, USA) between 100 and 1300 nm. lEV sizes were analyzed using 405 nm violet side scatter. Data analysis was performed using FlowJo software (version 10 for Windows) (FlowJo, LLC, Ashland, OR, USA).

### Western blot analysis

The amounts of total protein from lEV samples were determined with a bicinchoninic acid assay kit (Pierce, Thermo Scientific, Rockford, IL, USA), according to the manufacturer’s instructions. Western blot analysis was used to determine the EV protein markers, cardiomyocyte marker and EV purity in lEV samples. All samples were adjusted to 30 μg of total protein before mixing with reducing sample buffer and loaded into 10% SDS–polyacrylamide gel electrophoresis. Then, proteins were transferred to a PVDF membrane (Amersham Hybond PVDF Membrane, GE Healthcare Life Sciences, Freiburg, Germany). Membrane was blocked with 5% w/v non-fat milk in TBST buffer and then incubated with primary antibodies; anti-CD63, anti-Alix, anti-Apolipoprotein A (Abcam, Cambridge, MA, USA) and anti-Caveolin 3 (Invitrogen, Thermo Fisher Scientific, Waltham, MA USA) overnight at 4 °C. After washing, membranes were incubated with HRP linked goat anti-rabbit IgG (Abcam Cambridge, MA, USA) for 1 h at room temperature. Chemiluminescent detection was performed using Clarity Western ECL Substrate (Biorad Laboratories, Inc, Hercules, CA, USA). Protein bands were visualized by ImageQuant LAS 4000 (GE Healthcare Life Sciences).

### Quantitative reverse transcription polymerase chain reaction (RT-qPCR) array

Twelve AF patients and 12 non-AF controls were pooled into 4 samples in each group (3 subjects per 1 sample). Total lEV-RNA was extracted using TRIzol LS® Reagent (Life Technologies, Carlsbad, CA, USA) following the manufacturer’s protocol. RNA extraction efficiency was checked via three spike-in controls: UniSp2, UniSp4, and UniSp5. Total RNA was quantified using a Qubit™ RNA HS Assay Kit and a Qubit® 2.0 Fluorometer (both Life Technologies). Total RNA and small RNA profiles were investigated using an Agilent 2100 Bioanalyzer system with an Agilent RNA 6000 Pico Kit and an Agilent Small RNA Kit, respectively (Agilent Technologies, Santa Clara, CA, USA). Reverse transcription was performed using a miRCURY LNA™ Universal RT microRNA PCR (Qiagen, Hilden, Germany) using cel-miR-39-3p and UniSp6 as internal control. Expression of lEV- miRNA profiles was measured using miRCURY LNA miRNA Serum/Plasma Focus PCR Panels (Qiagen) containing the 179 most abundant miRNAs in circulation. RT-qPCR was performed using a CFX96™ Real-Time PCR Detection System (Bio-Rad Laboratories, Hercules, CA, USA) with a miRCURY LNA™ SYBR Green PCR Kit (Qiagen). All samples passed internal quality control (QC) checks for extraction, reverse transcription, qPCR efficiency, and hemolysis. The data analysis was performed on the QIAGEN web portal at the GeneGlobe Data Analysis Center (https://geneglobe.qiagen.com/us/analyze/). The results were reported as cycle threshold (Ct) values, which were normalized using the global Ct mean of expressed miRNAs method. lEV-miRNA expression was calculated as fold change relative to non-AF controls using the 2^−ΔΔCT^ method.

### Droplet digital polymerase chain reaction (ddPCR)

In the validation phase, lEV-miRNA expression was confirmed in 30 AF patients and 30 non-AF controls using a ddPCR™ system (Bio-Rad Laboratories). lEV-RNA concentration was measured using a NanoDrop™ 8000 Spectrophotometer (Thermo Fisher Scientific, Waltham, MA, USA). Two hundred nanograms of total RNA was reverse transcribed using a miRCURY LNA™ Universal RT microRNA PCR (Qiagen). One microliter of synthesized cDNA was added to a 20 μl PCR reaction mixture containing 10 μl of 2× EvaGreen Supermix (Bio-Rad Laboratories), 1 μl miRCURY LNA miRNA PCR primer (Qiagen), and 8 μl RNase-free H_2_O. Twenty microliters of ddPCR assay mixture was loaded into a disposable droplet generator cartridge (Bio-Rad Laboratories) with 70 μl of QX200 Droplet Generation Oil for EvaGreen. Detailed information about primers for miRNAs is presented in Additional file 1: Table S1. The cartridges were placed inside the QX200 Droplet Generator (Bio-Rad Laboratories), and then the droplet mixtures were transferred to a ddPCR™ 96-well PCR plate (Bio-Rad Laboratories). PCR amplification was performed using a C1000 Touch Thermal Cycler (Bio-Rad Laboratories). The thermal cycling conditions were, as follows: 95 °C for 5 min, 40 cycles of 95 °C for 30 s, and 54 °C for 1 min (ramping rate reduced to 2%), and three final steps at 4 °C for 5 min, 90 °C for 5 min, and 4 °C indefinite hold. A no template control (NTC) was included in every assay. The PCR-positive and negative droplets were read using a Q×200 Droplet Reader (Bio-Rad Laboratories). QuantaSoft software (Bio-Rad Laboratories) was used to quantitate the concentration of miRNAs, and the results are presented as the number of copies per microliter (no. copies/μl) of PCR reaction. Synthetic miRNA (Cel-miR-39-3p) was added into all samples to check RNA extraction efficiency.

### Bioinformatics analysis

MicroRNA regulation was analyzed using miRNA target gene prediction and pathway analysis. Diana-Tarbase version 8.0 (https://carolina.imis.athena-innovation.gr/diana_tools/web), which is a database of experimentally supported miRNA-gene interactions, was used to identify the target gene of the miRNAs miR-106b-3p, miR-590-5p, miR-339-3p, miR-378-3p, and miR-532-3p. The TargetScan miRNA target prediction tool (http://www.targetscan.org/vert_72/) was used to predict target genes of miR-328-3p. Prediction of miRNA function and pathway was performed using Diana-miRPath version 3 (http://snf-515788.vm.okeanos.grnet.gr/) with default settings. The miRNA target genes were categorized according to biological process, molecular function, and cellular component using the Gene Ontology (GO) (http://geneontology.org/) bioinformatics database. Functional pathways related to the cardiovascular system were identified by Kyoto Encyclopedia of Genes and Genomes (KEGG) pathways analysis. The threshold value was set at *p* < 0.05.

### Statistical analysis

Data were analyzed using PASW Statistics version 18 (SPSS, Inc., Chicago, IL, USA). Baseline characteristics compared between two groups was analyzed using chi-square test for categorical variables, and those results are reported as number and percentage. Unpaired t-test and Mann–Whitney U test were used to compare normally distributed (reported as mean ± standard deviation [SD]) and non-normally distributed continuous variables (reported as median and interquartile range [IQR]), respectively. Multivariate logistic regression analysis was performed to investigate for association between miRNA levels and AF. The results of logistic regression analysis are reported as odds ratio (OR) and 95% confidence interval (CI). Significant difference was defined as a *p*-value less than 0.05.

## Results

### Patient characteristics

The demographic, clinical, and treatment characteristics of the AF and non-AF groups in both the discovery phase and the validation phase are shown in Table 1. In discovery cohort, taking a beta-blocker (66.7% *vs.* 16.7%, *p* = 0.01) and a warfarin (83.3% vs. 0%, *p* =  < 0.001) were significantly higher among AF patients than among non-AF controls. In the validation cohort, the mean diastolic blood pressure of AF patients was significantly higher than in non-AF controls (79.1 ± 12.0 *vs.*72.7 ± 10.1, *p* = 0.03). The proportion of dyslipidemia was significantly lower in AF patients than non-AF controls (63.3% *vs.* 90%, *p* = 0.02), whereas congestive heart failure (30.0% *vs.* 0%, *p* = 0.001) and stroke (13.3% *vs.* 0%, *p* = 0.04) were significantly higher in AF patients than in non-AF controls. Taking a beta-blocker (60.0% *vs.* 23.3%, *p* = 0.004) was significantly higher among AF patients than among non-AF controls.

**Table 1:** Demographic and clinical characteristics of populations in the discovery and validation study

Characteristics	Discovery study			Validation study		
	AF (n = 12)	Non-AF (n = 12)	P value	AF (n = 30)	Non-AF (n = 30)	P value
Age, years (mean ± SD)	71.9 ± 6.0	69.0 ± 4.1	0.33	68.9 ± 9.1	69.2 ± 8.0	0.88
Male n, (%)	6 (50.0)	6 (50.0)	1.00	25 (83.3)	23 (76.7)	0.52
BMI (kg/m^2^)	24.1 ± 1.2	26.2 ± 1.3	0.22	26.4 ± 3.4	25.8 ± 3.0	0.47
Systolic BP (mmHg)	134.5 ± 6.0	134.0 ± 5.4	0.95	136.8 ± 19.9	132.8 ± 14.3	0.38
Diastolic BP (mmHg)	78.4 ± 4.9	74.7 ± 3.2	0.53	79.1 ± 12.0	72.7 ± 10.1	0.03
Heart rate (beats per min)	75.8 ± 3.6	86.8 ± 4.2	0.06	74.2 ± 15.3	75.4 ± 9.6	0.70
Medical history n, (%)						
Hypertension	11 (91.7)	9 (75.0)	0.27	23 (76.7)	21 (70.0)	0.56
Diabetes mellitus	5 (41.7)	4 (33.3)	0.67	9 (30.0)	11 (36.7)	0.58
Dyslipidemia	10 (83.3)	9 (75)	0.62	19 (63.3)	27 (90.0)	0.02
Congestive heart failure	1 (8.3)	0(0.0)	0.31	9 (30.0)	0 (0.0)	0.001
Stroke	1 (8.3)	0(0.0)	0.31	4 (13.3)	0 (0.0)	0.04
Bleeding history	1 (8.3)	0(0.0)	0.31	5 (16.7)	2 (6.7)	0.23
Smoking status						
Ex-smoker	5 (41.7)	1 (8.3)	0.06	17 (56.7)	12 (40.0)	0.33
Current smoker	0 (0.0)	0 (0.0)		1 (3.3)	3 (10.0)	
Never smoke	7 (58.3)	11 (91.7)		12 (40.0)	15 (50.0)	
Medications n, (%)						
Beta-blocker	8 (66.7)	2 (16.7)	0.01	18 (60.0)	7 (23.3)	0.004
Calcium channel blocker	6 (50.0)	4 (33.3)	0.41	9 (30.0)	11 (36.7)	0.58
ACE inhibitors or ARB	6 (50)	3 (25.0)	0.21	13 (43.3)	14 (46.7)	0.80
Statin	7 (58.3)	9 (75.0)	0.39	18 (60.0)	24 (80.0)	0.09
Warfarin	10 (83.3)	0 (0.0)	< 0.001	18 (60.0)	0 (0.0)	< 0.001
Aspirin	0 (0.0)	0 (0.0)	N/A	1 (3.3)	5 (16.7)	0.09

Data presented as mean ± SD for continuous variables and frequency (percentage) for categorical variables

*AF* atrial fibrillation, *BMI* body mass index, *BP* blood pressure, *ACE* angiotensin-converting enzyme, *ARB* angiotensin receptor blockers

### Characterization of large extracellular vesicles (lEVs) and lEV-derived RNA (lEV-RNA)

**Fig. 1:**
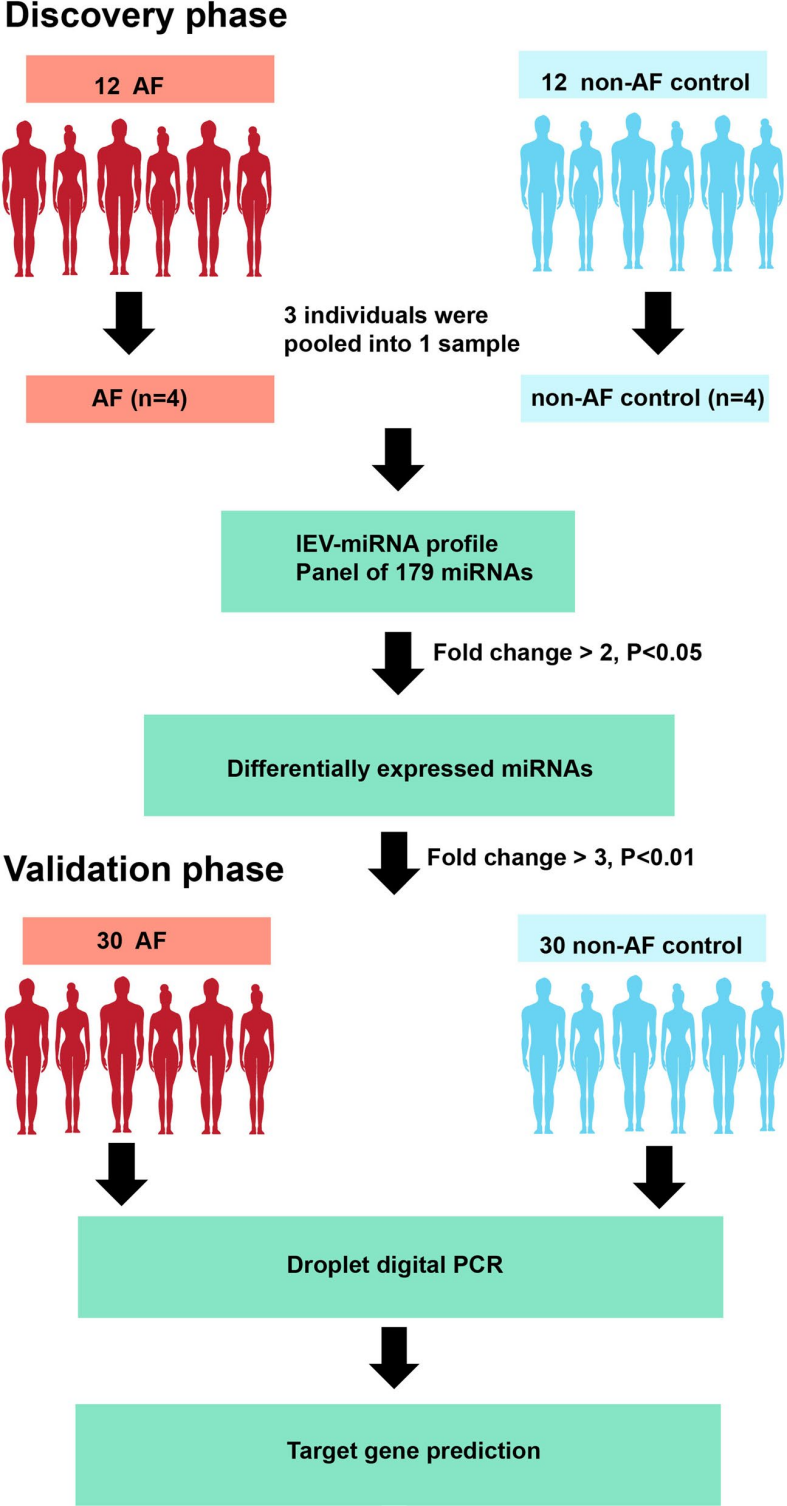
Flow diagram of the study protocol for the discovery and validations phases of this study

**Fig. 2:**
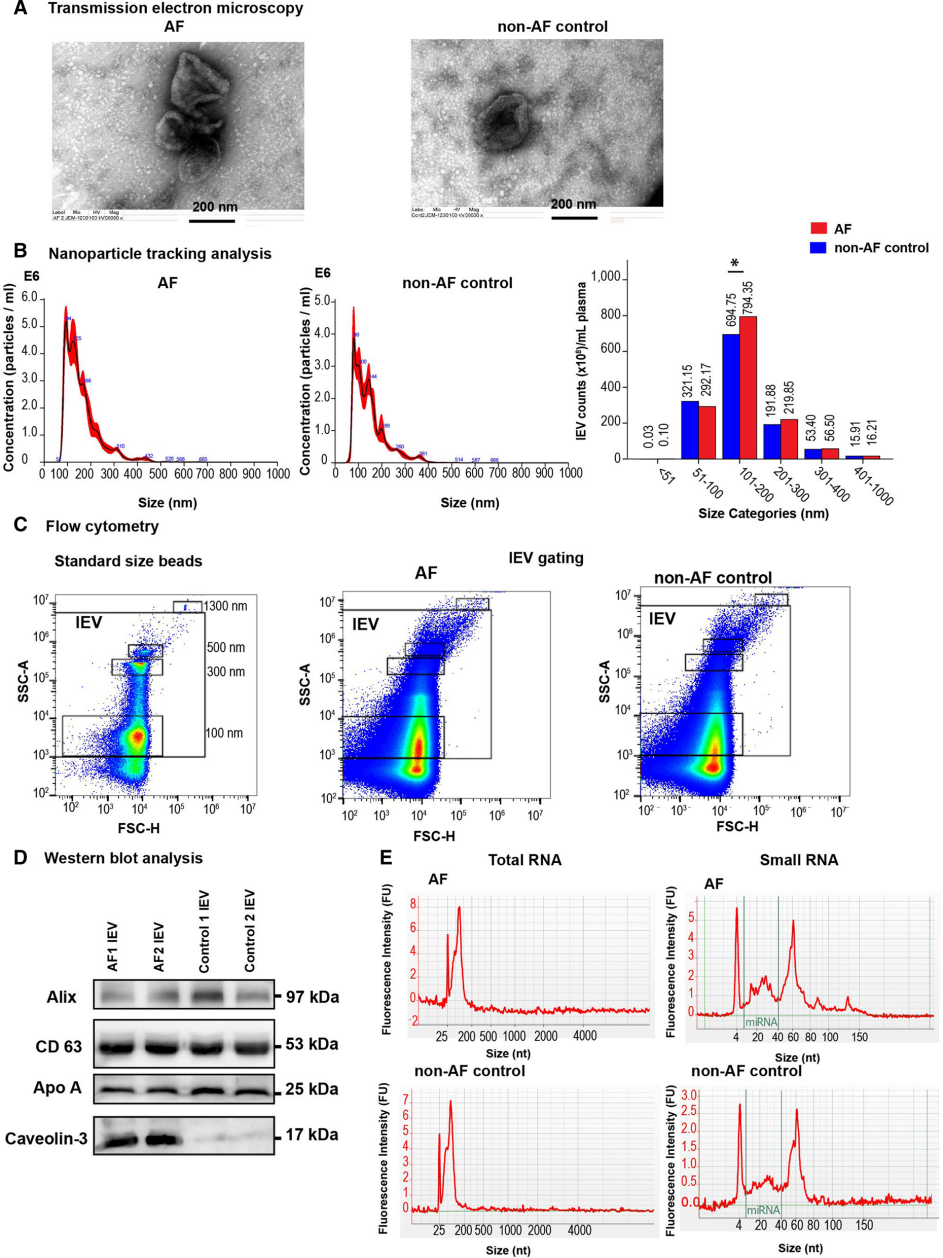
Characterization of large extracellular vesicles (lEVs) and lEV-RNA. Plasma lEVs from atrial fibrillation (AF) patients and non-AF controls were characterized by transmission electron microscopy (TEM), nanoparticle tracking analysis (NTA), flow cytometry and Western blot analysis. **A** lEVs were visualized by TEM (scale bar: 200 nm, 50,000 × magnification). **B** NTA demonstrating size distribution of lEVs and comparison of concentrations across to size categories of lEVs between AF patients (n = 42) and non-AF controls (n = 42). Bar graph presented as median values. **C** Flow cytometry showing lEV gating strategy following standard size bead between 100 and 1300 nm. **D** Western blot analysis of EV markers (Alix, and CD63), contaminant lipoprotein marker (Apo A) and cardiomyocyte marker (Caveolin-3) in lEV samples from AF patients and non-AF controls. (30 µg protein per lane). **E** lEV-RNA and lEV-small RNA profiles were determined by using a bioanalyzer. *p < 0.05, compared with non-AF controls

A flow chart demonstrating the two-phase design of this study is shown in Fig. 1. Plasma lEVs were isolated from AF patients and non-AF controls, and then were characterized by TEM, NTA, flow cytometry, and Western blot analysis. Size and morphology of lEVs were determined by TEM and NTA. lEVs presented cup shaped vesicles (Fig. 2A) with a size ranging from 50–1000 nm (Fig. 2B). There were no significant differences in total number, mean size, or mode size of lEVs between two groups (Additional file 2: Figure S1). To clarify the relationship between size distribution and concentration of lEVs, we classified lEV size into subcategories. The number of lEVs at the size of 101–200 nm in AF patients was significantly greater than non-AF controls (794.35 [603.49–1,013.80] × 10^8^/mL *vs.* 694.75 [559.93–822.63] × 10^8^/mL; *p* = 0.038), whereas there were no significance differences in other size categories between AF patients and non-AF controls (Fig. 2B). The results of flow cytometry were similar, with lEV diameter ranging from 100 to 1000 nm in size both AF patients and non-AF controls (Fig. 2C). Western blot analysis presented the expression of EV markers (CD63 and Alix) in lEV samples from two groups. Co-isolate contamination was evaluated the presence of apolipoprotein A (Apo A) expression, which is no difference between AF patients and non-AF controls. To address whether lEVs are released from cardiomyocytes, we determined the expression of Caveolin-3, which is skeletal muscle and cardiomyocyte marker. Interestingly, expression of Caveolin-3 in lEVs from AF patients was higher than those from non-AF controls (Fig. 2D), which suggested that cardiomyocytes are one of the sources of lEVs. To confirm whether RNA and miRNA truly existed in lEV samples, we extracted lEV-RNA and measured the RNA profile using automated electrophoresis. lEV-RNAs showed high peaks at below 200 bases with no bands of 28S or 18S ribosomal RNA, and these findings were confirmed using small RNA chips. The results showed that lEV-RNA was enriched relative to the size of miRNAs (10–40 bases) in both AF patients and non-AF controls (Fig. 2E).

### Discovery phase: identification of differentially expressed lEV-miRNAs between AF patients and non-AF controls

**Fig. 3:**
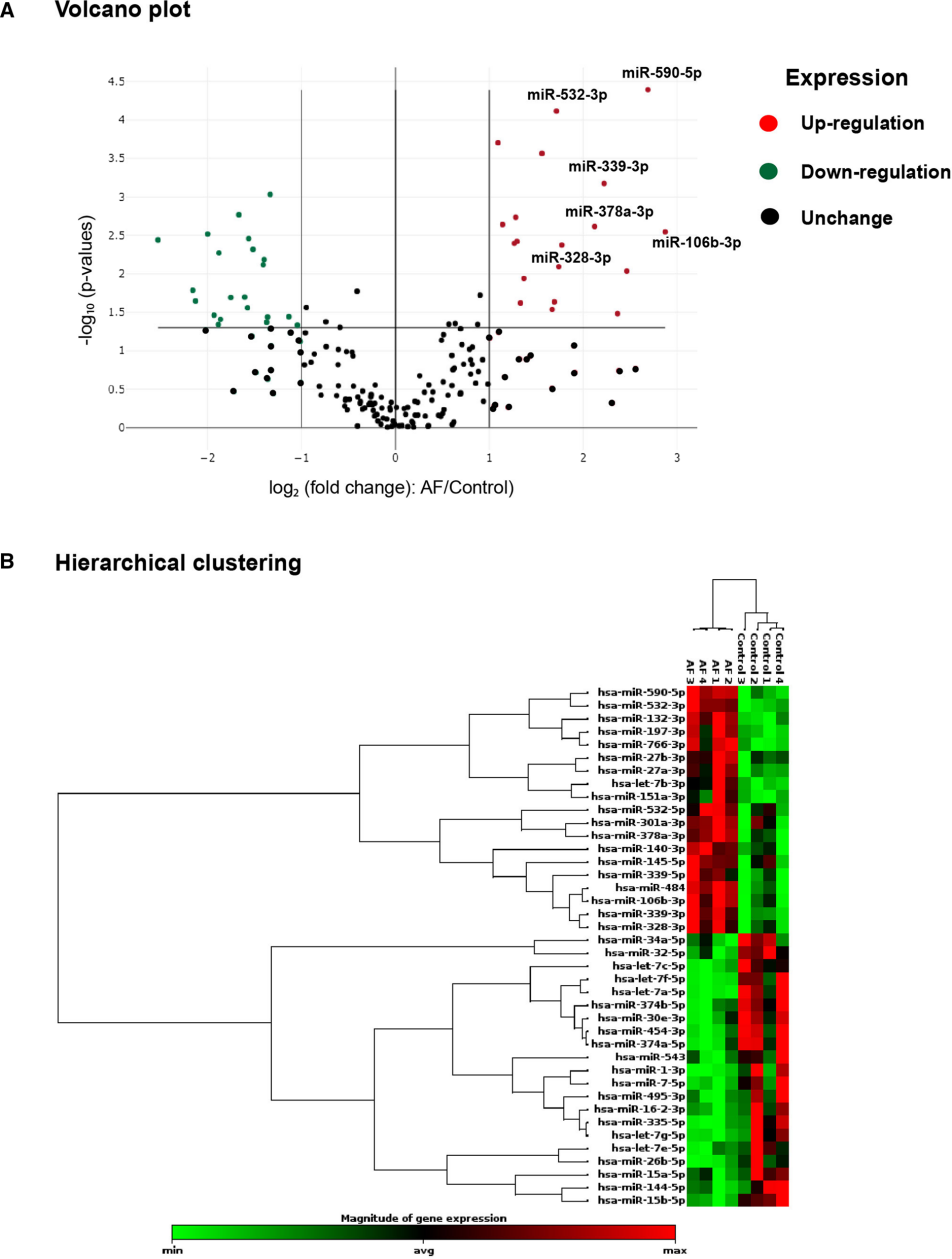
Large extracellular vesicles microRNA (lEV-miRNA) expression profile. **A** Volcano plot illustrates lEV**-**miRNAs that were significantly changed by more than two-fold (x-axis) with a *p*-value less than 0.05 (y-axis) between atrial fibrillation (AF) patients and non-AF controls. The red dots indicate significantly upregulated miRNAs. The green dots indicate significantly downregulated miRNAs. **B** Hierarchical clustering of differentially expressed lEV-miRNAs between AF patients (n = 4) and non-AF controls (n = 4). MicroRNA clustering tree shown on the left, and sample clustering tree shown on the right. Red indicates miRNAs with relatively high expression, and green indicates miRNAs with relatively low expression

To investigate the difference in lEV-miRNA profiles between AF patients and non-AF controls, 179 miRNAs were investigated from 4 pooled samples from both groups using a Serum/Plasma Focus microRNA PCR panel. The expression levels of 179 miRNAs are demonstrated in Additional file 3: Table S2. MicroRNA expression with more than a two-fold increase or decrease and a *p*-value less than 0.05 was considered to be a significant difference between groups. Differentially expressed lEV- miRNAs are shown as hierarchical clustering and a volcano plot in Fig. 3. Volcano plot shows the 19 significant upregulated lEV-miRNAs (red dots), and the 21 significant downregulated lEV-miRNAs (green dots) in AF patients compared to non-AF controls (Fig. 3A). Hierarchical clustering illustrates the most significant differentially expressed lEV-miRNAs in AF patients and non-AF controls (Fig. 3B). Fold changes in differentially expressed lEV-miRNAs are summarized in Additional file 4: Table S3. We selected the 6 most highly expressed lEV-miRNAs. All of those had more than a threefold increase and a *p*-value less than 0.01, and all of these 6 lEV-miRNAs have been reported to be involved in cardiovascular diseases, including miR-106b-3p (fold change [FC]: 7.33, *p* = 0.003), miR-590-5p (FC: 6.45, *p* = 0.00004), miR-339-3p (FC: 4.67, *p* = 0.0007), miR-378-3p (FC: 4.35, *p* = 0.002), miR-328-3p (FC: 3.34, *p* = 0.008), and miR-532-3p (FC: 3.28, *p* = 0.00008).

### Validation of differentially expressed lEV-miRNAs using dd-PCR

**Fig. 4:**
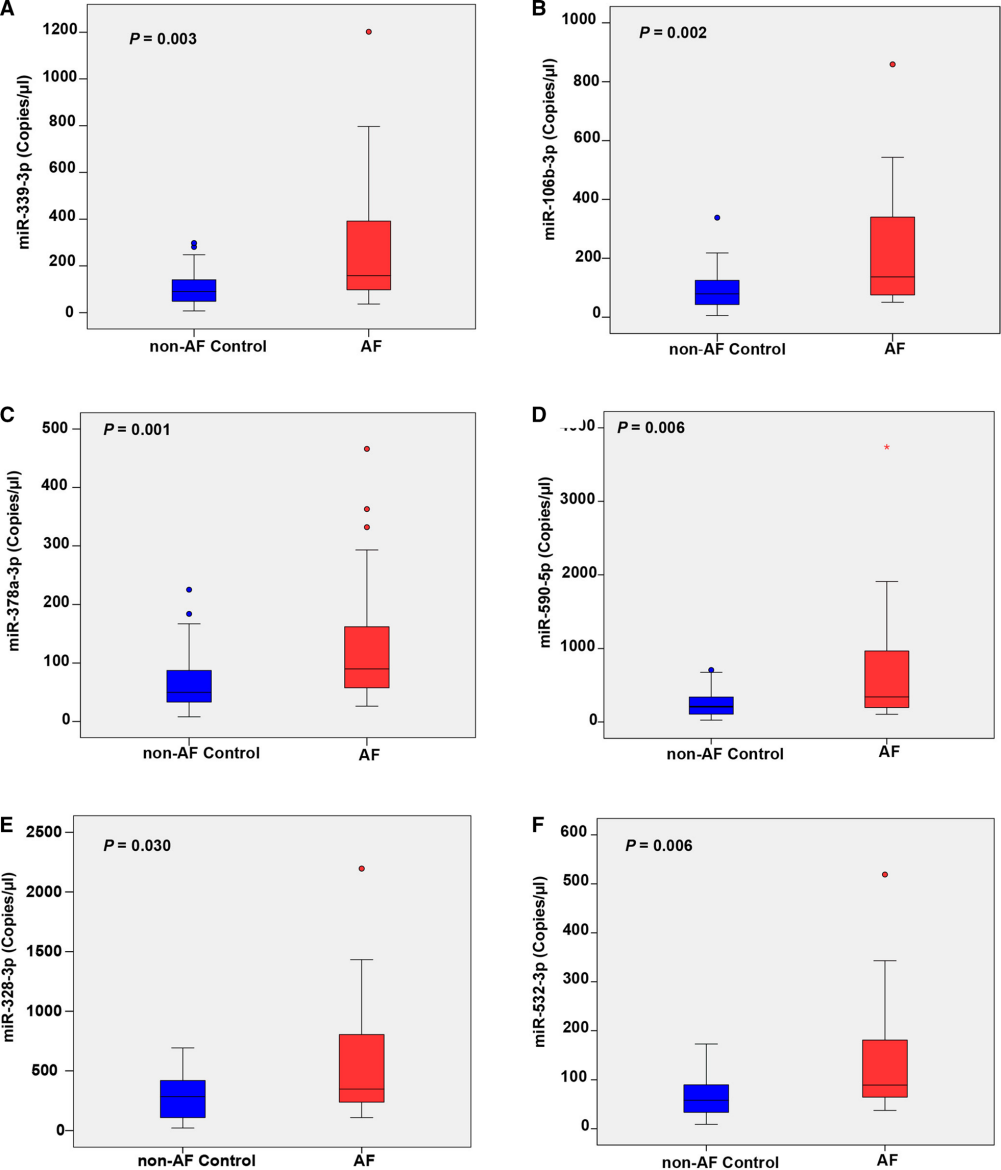
Absolute quantification of 6 differentially expressed lEV-miRNAs by droplet digital PCR. The expression levels of **A** miR-339-3p, **B** miR-106b-3p, **C** miR-378a-3p, **D** miR-590-5p, **E** miR-328-3p, and **F** miR-532-3p were significantly upregulated in atrial fibrillation (AF) patients (n = 30) relative to those in non-AF controls (n = 30). The levels of miRNA are presented as copies/μl of PCR reaction. The box plots indicate median and interquartile range (Q1–Q3)

**Fig. 5:**
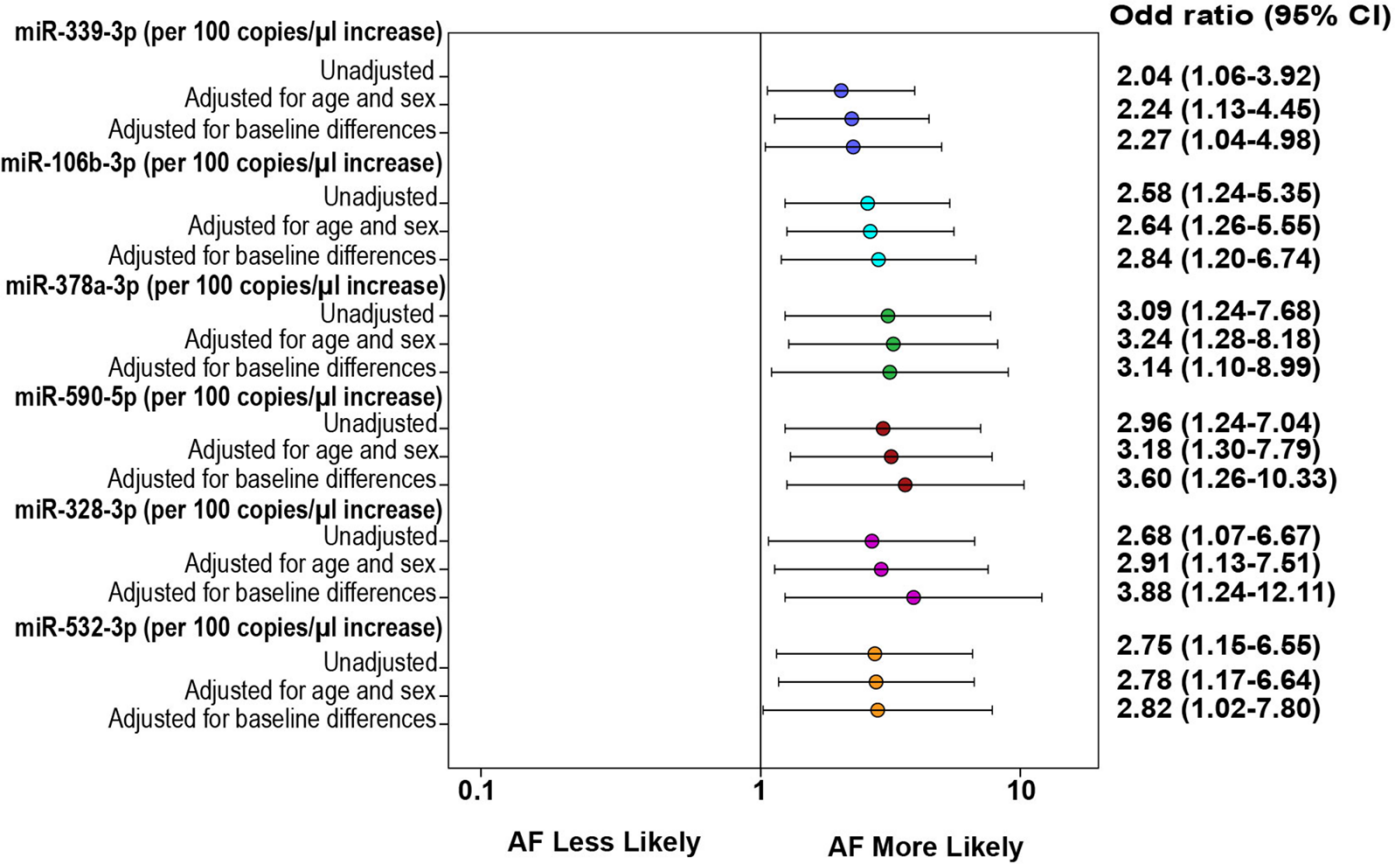
Forest plot showing odd ratios for association between 6 highly expressed lEV-microRNAs and atrial fibrillation. Baseline differences adjusted for diastolic blood pressure, dyslipidemia and beta blocker

To validate the levels of 6 highly expressed lEV-miRNAs in AF patients and non-AF controls, we performed absolute quantification using dd-PCR. Independent sets of AF patients and non-AF controls (n = 30) were investigated in the validation phase. Consistent with the findings of the discovery phase, the median (interquartile range [IQR]) expression levels were significantly higher in AF than in non-AF controls, as follows: miR-106b-3p (137.00 [73.08–347.25] copies/μl *vs.* 79.00 [42.40–125.50] copies/μl *p* = 0.002); miR-590-5p (341.50 [194.25–993.50] copies/μl *vs.* 208.00 [100.58–340.75] copies/μl, *p* = 0.006); miR-339-3p (158.00 [95.25–400.75 copies/μl *vs.* 90.25 [48.40–142.00] copies/μl, *p* = 0.003); miR-378a-3p (89.80 [56.85–167.50] copies/μl *vs.* 49.70 [32.55–87.88] copies/μl, *p* = 0.001); miR-328-3p (348.00 [37.25–842.00] copies/μl *vs.* 284.00 [105.78–435.25] copies/μl, *p* = 0.030); and, miR-532-3p (89.25 [63.55–186.75] copies/μl *vs.* 58.20 [33.33–94.95] copies/μl, *p* = 0.006), respectively (Fig. 4). Moreover, there were no significant differences of the 6 highly expressed miRNA between patients with new or paroxysmal AF and persistent or permanent AF as presented in Additional file 5: Table S4. Logistic regression analysis showed that per 100 copies/μl increase in the levels of miR-378-3p (odds ratio [OR]: 3.09, 95% confidence interval [CI] 1.24–7.68; *p* = 0.015), miR-339-3p (OR: 2.04, 95% CI 1.06–3.92; *p* = 0.032), miR-106b-3p (OR: 2.58, 95% CI 1.24–5.35; *p* = 0.011), miR-328-3p (OR: 2.68, 95% CI 1.07–6.67; *p* = 0.035), miR-532-3p (OR: 2.75, 95% CI 1.15–6.55; *p* = 0.022), and miR-590-5p (OR: 2.96, 95% CI 1.24–7.04; *p* = 0.014) to be positively associated with AF. After adjustment for age, sex, and baseline differences all 6 highly expressed lEV-miRNAs had a slightly higher odds ratio, as shown in the forest plot in Fig. 5.

### Potential target prediction for differentially expressed lEV-miRNAs

**Fig. 6:**
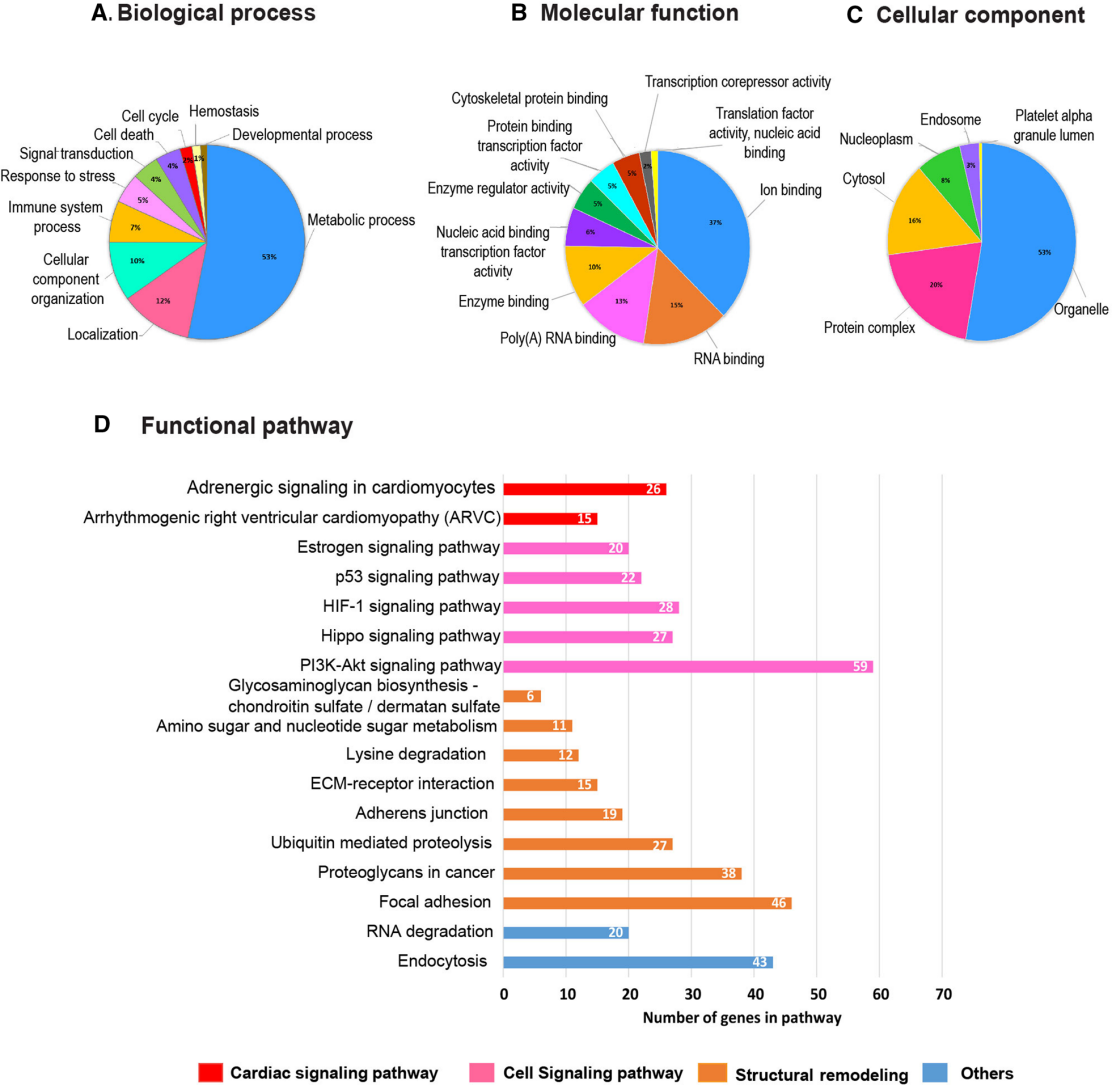
Bioinformatics analysis of putative microRNA (miRNA) target genes. Gene Ontology (GO) and Kyoto Encyclopedia of Genes and Genomes (KEGG) pathways analysis of target genes regulated by 6 highly expressed lEV-miRNAs in patients with atrial fibrillation (AF). Top 10 GO enrichment of target genes based on the categories of **A** biological process, **B** molecular function, and **C** cellular component. **D** Functional pathways of 6 highly expressed lEV-miRNAs involved in AF pathophysiology

To study the roles of the 6 highly expressed lEV-miRNAs under AF pathological conditions, bioinformatics analysis was performed. The target genes of 6 highly expressed lEV-miRNAs in AF patients are presented in Additional file 6: Table S5. We categorized miRNA target genes according to their biological process, molecular function, and cellular component using GO analysis (Fig. 6). Concerning biological process, most target genes were found to play roles in metabolic process, localization and cellular component organization (Fig. 6A). Regarding molecular function roles, target genes were found to be mostly involved in ion binding, RNA binding, and poly(A) RNA binding (Fig. 6B). Lastly, cellular components of target proteins were predominantly located in organelle, protein complex, and cytosol (Fig. 6C). Functional analysis revealed that the 6 highly expressed lEV-miRNA targets may involve in AF pathophysiology, including cardiac signaling pathway (adrenergic signaling in cardiomyocytes, arrhythmogenic right ventricular cardiomyopathy), cell proliferation (Hippo signaling pathway, PI3K-Akt signaling pathway), cell apoptosis (p53 signaling pathway), oxygen homeostasis (HIF-1 signaling pathway), and structural remodeling (Focal adhesion, ECM-receptor interaction) (Fig. 6D).

## Discussion

This is the first study of lEV-miRNA profile in AF patients, and our results suggest several important implications. First, we found upregulation of 6 lEV-miRNAs to be significantly associated with AF and to reflect important pathophysiology of AF. Second, the expression of selected lEV-miRNAs was evaluated by ddPCR, which is a highly sensitive and accurate technique that sidesteps the need to use unsuitable reference genes, and that eliminates false positive and false negative results. This technique may assist in the development of lEV-miRNA platform for clinical application in the future.

Many studies have reported a relationship between circulating miRNAs and AF. Downregulation of miR-150 was shown to be associated with AF in several studies [21–24]. Decreased levels of plasma and atrial tissue miR-29b have been found in AF patients, which suggests its role in atrial fibrotic remodeling by targeting collagen-1A1 (COL1A1) [25]. Low level of whole blood miR-328 was reported to be associated with prevalent AF [22], whereas high level of plasma miR-328 was found in AF patients, especially plasma from the left atrial appendage [26]. Due to the inconsistent results and unrelated levels of miRNAs between plasma and tissue, it has been suggested that circulating miRNAs may not useful biomarkers for AF [23]. In contrast, extracellular vesicle (EV)-derived miRNAs are potentially attractive biomarkers since they are more specific and reflect the pathogenesis of AF. Upregulation of exosomal miRNAs (miR-103a, miR-107, miR-320d, miR-486, and let-7b) has been demonstrated in patients with persistent AF compared with supraventricular tachycardia controls, which suggests that these miRNAs are involved in atrial function and structure, oxidative stress, and fibrosis pathways [14]. Wei, et al. found the expression levels of exosomal miR-92b-3p, miR-1306-5p, and let-7b-3p to be significantly increased in AF patients compared with normal sinus rhythm [13]. Another study showed exosomal miRNAs (miR-438-5p, miR-142-5p, miR-223-3p, and miR-223-5p) to be related to AF. Multivariate logistic analysis suggests that miR-483-5p is independently correlated with AF [15]. Epicardial fat-derived EVs (eFat-EVs) from patients with AF have been shown the upregulation of profibrotic miR-46b, whereas antifibrotic miR-33a and 29a are downregulated, compared to those without AF, which are associated with the stimulation of extensive myocardial fibrosis in the heart rats [27].

Fibrillating atria in AF has been shown to promote more myolysis, nuclear alteration, and apoptosis, which related to activation of programmed cell death via strongly upregulated CASP-3 expression and downregulated BCL-2 expression [28, 29]. Cell activation or apoptosis contributes to increased membrane permeability and remodeling, which subsequently results in lEV generation [30]. Several mechanisms in AF have been described as potent inducers of apoptotic cell death, such as high ventricular heart rate, low or oscillatory shear stress, stretch, hypoxia, inflammation, and oxidative stress, and all of these mechanisms can promote lEV generation [31]. Therefore, we hypothesized that the content within lEVs, especially miRNAs, may relate to the pathophysiology of AF.

There were no significant differences in total number, mean size and mode size of lEVs between AF patients and non-AF controls, suggesting that it may result from non-EV protein contamination. The relationship between lEV size and concentration was deeply investigated, which concentration of lEVs at the size of 101–200 nm in AF patients was significantly higher than non-AF controls. Elevated levels of this size range may result from the pathophysiology of AF that stimulates the mechanism of lEV generation [32]. For example, acute induction of AF activates platelets within minutes and significantly increases the expression of P-selectin on both platelets and platelet-derived lEVs [33]. The increase of circulating procoagulant lEVs might reflect a hypercoagulable state that could contribute to atrial thrombosis and thromboembolism. Mechanical stretch has been linked to Ca^2^ + overload in cardiomyocytes in AF [31, 34]. Increasing of intracellular Ca^2+^ activates cytoskeleton cleavage through calpain and caspase activation, leading to membrane remodeling ultimately results in lEVs shedding. Our previous study showed that circulating lEVs are released from platelets, endothelial cells, leukocytes and red blood cells [35]. However, more than 45% of lEVs in AF patients were not able to be characterized their cellular origins. In this study showed increased expression of Caveolin-3 in lEV samples from AF patients. These results revealed that cardiomyocytes are one of the sources of lEVs that may relate to an increase in lEV level at the size of 101–200 nm in AF patients.

lEV-miRNA profiling analysis revealed 19 significantly upregulated miRNAs, and 21 significantly downregulated miRNAs in AF patients compared to non-AF controls. The levels of six highly expressed miRNAs (miR-339-3p, miR-106b-3p, miR-378a-3p, miR-590-5p, miR-328-3p, and miR-532-3p) were confirmed with absolute quantification in our validation cohort, and logistic regression analysis showed the elevated levels of these miRNAs to be significantly associated with AF. Moreover, bioinformatics analysis demonstrated these 6 highly expressed miRNAs to likely be involved in arrhythmogenesis, cell apoptosis, cell proliferation, oxygen hemostasis, and structural remodeling—all of which are processes implicated in the pathogenesis of AF.

An abundance of evidence suggests that miRNAs may be directly or indirectly involved in AF by modulating atrial electrical remodeling (miR-328-3p, miR-106b-3p) and structural remodeling (miR-590-5p). miR-328-3p regulates particular genes involved in inflammation, myocyte depolarization (CACNA1C and CACNB1), vascular function (ABCG2), and cellular aging (H2AFX) [36–39]. Elevation of miR-328 level in left atrial tissue has been found in both canine AF models and AF patients with rheumatic heart disease. Overexpression of miR-328 in a canine model of AF diminished L-type Ca^2^ + current and shortened atrial action potential duration – both of which increase vulnerability to AF [40]. Previous reports have been suggested that many miRNAs promote atrial structural remodeling and fibrosis, including miR-328, miR-31, miR-1, miR-146b and miR-21, they are also detected in the circulation of various cancer patients. These miRNAs have been hypothesized that may relate to the high incidence of AF in cancer patients via their repressive effects in the genes that control cardiac arrhythmias [41].

Chiang et al. reported the miR-106b-25 cluster (miR-25, miR-93, and miR-106b) to be mediators of electrical remodeling, and downregulation of the miR-106b-25 cluster has been found in the atria of patients with AF. Loss of the miR-106b-25 cluster leads to upregulation of ryanodine receptor type-2 (RyR2) protein levels and pro-arrhythmic sarcoplasmic reticulum Ca^2^ + -release, which are associated with increased AF susceptibility [42]. Nicotine use is associated with downregulation of miR-590-5p, which partly explains the upregulation of TGF-β1 and TGF-βR2 proteins in the right atrial appendage of human and canine AF models. These two proteins promote the production of collagens in the myocardium, which was found to relate to the development of myocardial fibrosis, and the subsequent induction of atrial structural remodeling and fibrillation [43].

miR-378a-3p, which is the most abundant miRNA in skeletal muscles, including cardiac muscles, was reported to be involved in anti-apoptosis [44], angiogenesis [45], anti-fibrosis [46], and anti-hypertrophy [47]. Downregulation of miR-378a-3p has been found in the right atrial appendages (RAA) and left atrial appendages (LAA) in AF patients with rheumatic mitral valve disease [48]. It is, therefore, possible that downregulation of miR-378a-3p partly promotes cardiac apoptosis, fibrosis, and hypertrophy in AF. Two studies reported the pro-apoptotic role of miR-532-3p via suppression of apoptosis repressor with caspase recruitment domain (ARC) [49, 50]. Upregulation of whole blood miR-339-3p has been observed in coronary heart disease [51]. Ming Tana, et al. reported that the levels of miR-339-3p increase in mouse plasma exosomes before thrombosis, and that they are enriched in thrombin-stimulated platelet-derived exosomes in vitro, which suggests association with platelet activation [52]. Many studies have shown high levels of platelet-derived MPs in AF compared to healthy controls, which suggests their potential involvement in platelet activation [35, 53, 54]. In the present study, the level of lEV-derived miR-339-3p was upregulated in AF patients, which may result from selective packaging of miR-339-3p from activated platelets into their lEVs during AF.

Upregulation of 6 lEV-miRNAs in AF patients may be involved in two main mechanisms. First, lEVs act as a mediator in cell–cell communication. Upregulation of miR-378a-3p, miR-590-5p, and miR-106b-3p in lEVs may reflect cardioprotective signaling for anti-arrhythmia, anti-fibrosis, anti-apoptosis and anti-hypertrophy, which means that upregulation of these lEV-miRNAs could attenuate the pathogenesis of AF. Second, upregulation of miR-328-3p, miR-532-3p, and miR-339-3p may be involved in the generation of lEVs following cardiomyocyte activation or apoptosis, which could release high levels of apoptosis-related miRNAs in lEVs into blood circulation.

## The strengths and limitations

The strengths of this study included the in-depth analysis of lEV-miRNA profile and the possible roles of lEVs-miRNAs in AF. Our previous study has been shown high levels of lEVs in AF patients, especially platelet and endothelial-derived lEVs. This is the first study that explored the contents of lEV, particular miRNAs, which could be the main players in cell to cell communication. The large number of lEV-miRNAs were investigated by using high-throughput quantitative reverse transcription polymerase chain reaction (RT-qPCR) array for screening of lEV-miRNAs candidates. The validation phase was performed with high sensitivity technique (ddPCR), which resolved problems of low abundance lEV-miRNAs and the lack of suitable reference gene. Moreover, we demonstrated the increased expression of cardiomyocyte markers (Caveolin-3) in lEVs from AF patients.

However, our study has some limitations that should be considered. Small sample size in high-throughput miRNA screening may obtain some false positive and false negative results. However, confirmation in the larger population must be performed. Platforms of qRT-PCR array can only identify abundance and known miRNAs in plasma and serum, the data of low abundance and new miRNAs are limited. To resolve this limitation, platforms of high throughput next generation sequencing allows both discovery of new miRNAs and confirmation of known miRNAs. The selection of candidate miRNAs was performed based on remarkable differences in the fold changes and information in previous reports of CVDs. The other differentially expressed miRNAs, which were not selected for further analysis, may also play importance role in AF. We did not focus on AF subtypes and AF progression. To address for this limitation, lEV-miRNA profiling should be performed in a larger cohort that includes an appropriate proportion of each AF subtype. Data from a study with that design may yield important biomarkers that indicate AF progression. The results of our study can only suggest the possible roles of selected miRNAs in AF from bioinformatics analysis. As such, confirmation via functional study is needed. Further research should confirm the relationship between lEV-miRNAs and mRNA target genes in an in vitro or in vivo AF model. In addition, EVs loaded with miRNAs is emerging as a new approach to AF therapy [55]. Data from this study will be the useful for promoting this type of therapeutic application in AF.

## Conclusion

Expression levels of lEV-miRNAs (miR-339-3p, miR-106b-3p, miR-378a-3p, miR-590-5p, miR-328-3p, and miR-532-3p) were significantly upregulated in patients with AF compared to non-AF controls. These lEV-miRNAs may involve in arrhythmogenesis, cell apoptosis, cell proliferation, oxygen hemostasis, and structural remodeling in AF, which reflect the pathophysiology of AF. This study reports new findings that suggest roles of lEV-miRNAs in AF and may provide information to apply lEV-miRNAs as the novel biomarkers for diagnosis or monitoring the patients with high risk of AF.

## Supplementary Information

**Additional file 1: Table S1.** MiRCURY LNA™ polymerase chain reaction (PCR) primers used for ddPCR.

**Additional file 2: Figure S1.** The (A) number, (B) mean size, and (C) mode size of IEVs compared between atrial fibrillation (AF) patients (n=42) and non-AF controls (n=42).

**Additional file 3: Table S2.** Profiling of 179 miRNA expression in AF patients compared to non-AF controls.

**Additional file 4: Table S3. **A list of differentially expressed lEV-miRNAs in atrial fibrillation patients.

**Additional file 5: Table S4. **MiRNA levels of AF patients in the validation study comparing new or paroxysmal versus persistent or permanent.

**Additional file 6: Table S5.** Putative targets of 6 highly expressed lEV miRNAs in atrial fibrillation (AF) patients.

## Data Availability

All data generated or analysed during this study are included in this published article and its Additional files.
