# Supplementary material for: Increased expression of six-large extracellular vesicle-derived miRNAs signature for nonvalvular atrial fibrillation
Source: J Transl Med. 2022 Jan 3;20:4. doi: 10.1186/s12967-021-03213-6 (PMC8722074; doi:10.1186/s12967-021-03213-6)
Supplement: Supplementary file 1 — Additional file 1: Table S1. MiRCURY LNA™ polymerase chain reaction (PCR) primers used for ddPCR. [file 12967_2021_3213_MOESM1_ESM.docx]

**Table S1 MiRCURY LNA™ polymerase chain reaction (PCR) primers used for ddPCR**

| MicroRNA ID | MiRBase Accession | Target sequence | MiRCURY LNA miRNA PCR assay catalog number |
| --- | --- | --- | --- |
| hsa-miR-339-3p | MIMAT0004702 | ugagcgccucgacgacagagccg | YP00204160 |
| hsa-miR-328-3p | MIMAT0000752 | CUGGCCCUCUCUGCCCUUCCGU | YP00204364 |
| hsa-miR-378a-3p | MIMAT0000732 | ACUGGACUUGGAGUCAGAAGGC | YP00205946 |
| hsa-miR-590-5p | MIMAT0003258 | GAGCUUAUUCAUAAAAGUGCAG | YP00204222 |
| hsa-miR-106b-3p | MIMAT0004672 | CCGCACUGUGGGUACUUGCUGC | YP00204020 |
| hsa-miR-532-3p | MIMAT0004780 | CCUCCCACACCCAAGGCUUGCA | YP00204003 |
