# Supplementary figures and images for: Increased expression of six-large extracellular vesicle-derived miRNAs signature for nonvalvular atrial fibrillation
Source: J Transl Med. 2022 Jan 3;20:4. doi: 10.1186/s12967-021-03213-6 (PMC8722074; doi:10.1186/s12967-021-03213-6)

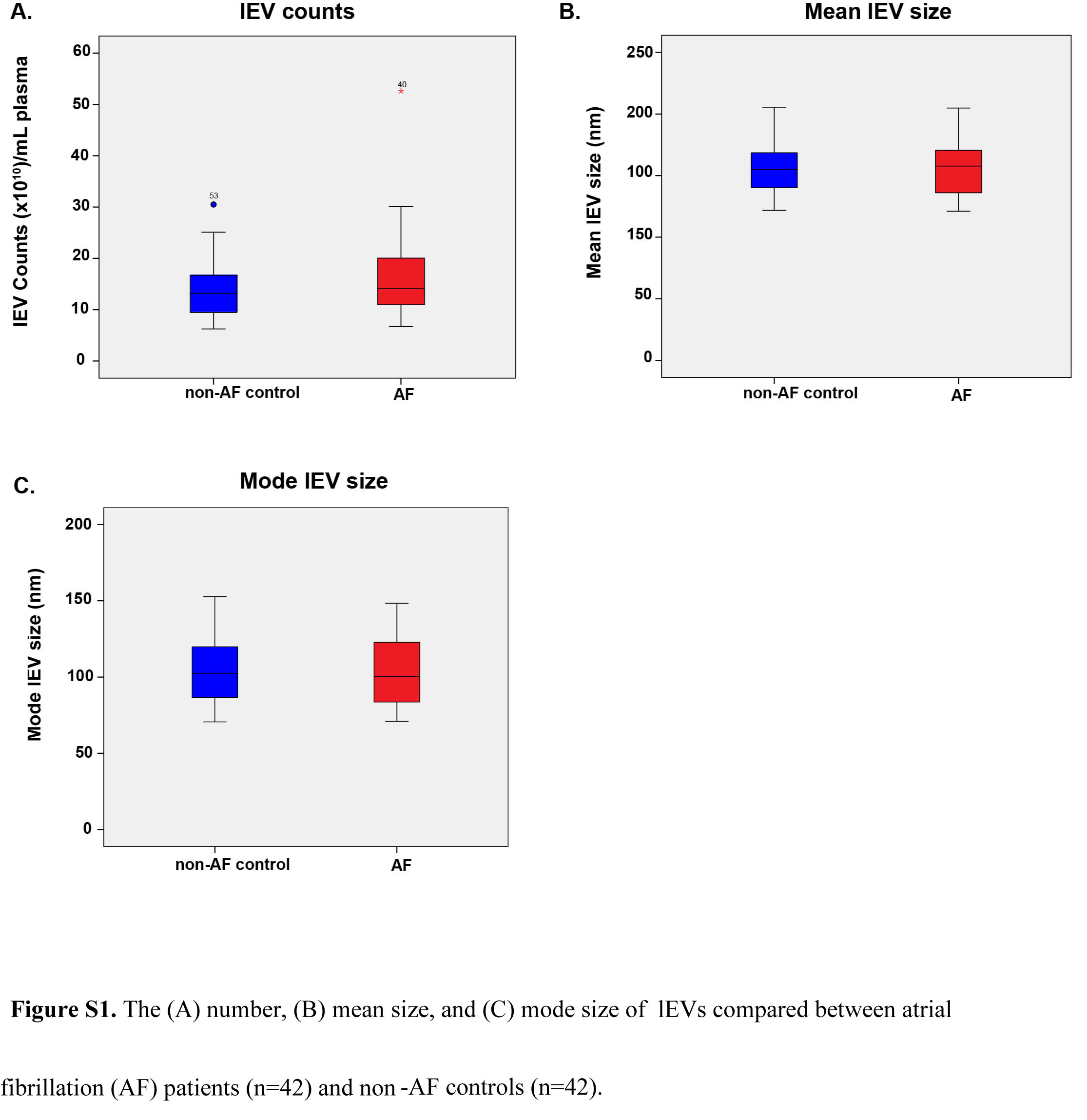

Supplement: Supplementary file 2 — Additional file 2: Figure S1. The (A) number, (B) mean size, and (C) mode size of IEVs compared between atrial fibrillation (AF) patients (n=42) and non-AF controls (n=42). [file 12967_2021_3213_MOESM2_ESM.jpg]
