# Supplementary material for: Increased expression of six-large extracellular vesicle-derived miRNAs signature for nonvalvular atrial fibrillation
Source: J Transl Med. 2022 Jan 3;20:4. doi: 10.1186/s12967-021-03213-6 (PMC8722074; doi:10.1186/s12967-021-03213-6)
Supplement: Supplementary file 4 — Additional file 4: Table S3. A list of differentially expressed lEV-miRNAs in atrial fibrillation patients. [file 12967_2021_3213_MOESM4_ESM.docx]

**Table S3.** A list of differentially expressed lEV-miRNAs in atrial fibrillation patients

| Up-regulated  miRNAs | Fold  Change | P-Value | Down-regulated  miRNAs | Fold  Change | P-Value |
| --- | --- | --- | --- | --- | --- |
| hsa-miR-27b-3p | 2.58 | 0.011505 | **hsa-let-7f-5p** | -5.77 | 0.003621 |
| hsa-miR-484 | 2.95 | 0.000272 | **hsa-miR-374b-5p** | -2.95 | 0.003484 |
| hsa-miR-145-5p | 3.18 | 0.029089 | **hsa-let-7e-5p** | -2.98 | 0.027683 |
| hsa-let-7b-3p | 2.41 | 0.004019 | **hsa-let-7a-5p** | -3.18 | 0.001712 |
| hsa-miR-197-3p | 2.21 | 0.002293 | **hsa-miR-26b-5p** | -4.37 | 0.022640 |
| hsa-miR-590-5p | 6.45 | 0.000040 | **hsa-miR-34a-5p** | -2.06 | 0.046548 |
| hsa-miR-301a-3p | 5.16 | 0.032991 | **hsa-miR-335-5p** | -4.46 | 0.016426 |
| hsa-miR-339-3p | 4.67 | 0.000670 | **hsa-let-7g-5p** | -3.37 | 0.020327 |
| hsa-miR-140-3p | 3.42 | 0.004225 | **hsa-miR-1-3p** | -3.81 | 0.034514 |
| hsa-miR-27a-3p | 2.45 | 0.003793 | **hsa-miR-32-5p** | -3.68 | 0.005356 |
| hsa-miR-328-3p | 3.34 | 0.008084 | **hsa-miR-15a-5p** | -2.19 | 0.036001 |
| hsa-miR-532-3p | 3.28 | 0.000077 | **hsa-let-7c-5p** | -4.00 | 0.003039 |
| hsa-miR-532-5p | 3.23 | 0.023222 | **hsa-miR-454-3p** | -2.64 | 0.006549 |
| hsa-miR-132-3p | 2.13 | 0.000198 | **hsa-miR-16-2-3p** | -2.57 | 0.036401 |
| hsa-miR-339-5p | 5.52 | 0.009220 | **hsa-miR-144-5p** | -2.58 | 0.042747 |
| hsa-miR-378a-3p | 4.35 | 0.002430 | **hsa-miR-374a-5p** | -2.86 | 0.004809 |
| hsa-miR-766-3p | 2.43 | 0.001839 | **hsa-miR-15b-5p** | -2.52 | 0.000929 |
| hsa-miR-151a-3p | 2.52 | 0.023991 | **hsa-miR-30e-3p** | -2.65 | 0.007645 |
| hsa-miR-106b-3p | 7.33 | 0.002851 | **hsa-miR-543** | -3.64 | 0.039134 |
|  |  |  | **hsa-miR-7-5p** | -3.04 | 0.020117 |
|  |  |  | **hsa-miR-495-3p** | -3.69 | 0.045678 |
