# Supplementary material for: Increased expression of six-large extracellular vesicle-derived miRNAs signature for nonvalvular atrial fibrillation
Source: J Transl Med. 2022 Jan 3;20:4. doi: 10.1186/s12967-021-03213-6 (PMC8722074; doi:10.1186/s12967-021-03213-6)
Supplement: Supplementary file 5 — Additional file 5: Table S4. MiRNA levels of AF patients in the validation study comparing new or paroxysmal versus persistent or permanent. [file 12967_2021_3213_MOESM5_ESM.docx]

**Table S4.** MiRNA levels of AF patients in the validation study comparing new or paroxysmal versus persistent or permanent

| **AF patients in Validation study** | | | |
| --- | --- | --- | --- |
| **Variables** | **New or paroxysmal AF (n=19)** | **Persistent or permanent AF (n=11)** | **P-value**  **(Mann-Whitney U test)** |
| miR-339-3p | 155.0 (82.5-231.0) | 161.0 (98.0-572.0) | 0.651 |
| miR-106b-3p | 146.0 (75.2-241.0) | 131.0 (66.7-390.0) | 0.715 |
| miR-378-3p | 117.0 (59.4-162.0) | 61.5 (55.2-184.0) | 0.621 |
| miR-590-3p | 431.0(159.0-894.0) | 295.0 (196.0-1529.0) | 0.846 |
| miR-328-3p | 345.0 (238.0-636.0) | 351.0 (235.0-1012.0) | 0.731 |
| miR-532-3p | 88.6 (60.7-119.0) | 100.0 (64.5-233.0) | 0.747 |

Data are shown as median and interquartile range (IQR)
